# Intracellular Reduction-Responsive Molecular Targeted Nanomedicine for Hepatocellular Carcinoma Therapy

**DOI:** 10.3389/fphar.2021.809125

**Published:** 2022-01-10

**Authors:** Lei Ding, Ping Zhang, Xu Huang, Kunmeng Yang, Xingkai Liu, Zhenxiang Yu

**Affiliations:** ^1^ Department of Hepatobiliary and Pancreatic Surgery, The First Hospital of Jilin University, Changchun, China; ^2^ Department of Respiration, The First Hospital of Jilin University, Changchun, China

**Keywords:** lenvatinib, polypeptide, reduction-responsive nanomedicine, hepatocellular carcinoma, molecular targeted therapy

## Abstract

The stimuli-responsive polymer-based platform for controlled drug delivery has gained increasing attention in treating hepatocellular carcinoma (HCC) owing to the fascinating biocompatibility and biodegradability, improved antitumor efficacy, and negligible side effects recently. Herein, a disulfide bond-contained polypeptide nanogel, methoxy poly(ethylene glycol)−poly(l-phenylalanine-*co*-l-cystine) [mPEG−P(LP-*co*-LC)] nanogel, which could be responsive to the intracellular reduction microenvironments, was developed to deliver lenvatinib (LEN), an inhibitor of multiple receptor tyrosine kinases, for HCC therapy. The lenvatinib-loaded nanogel (NG/LEN) displayed concise drug delivery under the stimulus of glutathione in the cancer cells. Furthermore, the intracellular reduction-responsive nanomedicine NG/LEN showed excellent antitumor effect and almost no side effects toward both subcutaneous and orthotopic HCC tumor-allografted mice in comparison to free drug. The excellent tumor-inhibition efficacy with negligible side effects demonstrated the potential of NG/LEN for clinical molecular targeted therapy of gastrointestinal carcinoma in the future.

## Introduction

Hepatocellular carcinoma (HCC), the most commonly diagnosed liver cancer, is the third most lethal malignancy in China, causing 4,221,000 deaths in 2015 (Chen et al., 2016). Although surgical resection is a mature treatment modality, the overall survival of 5 years is merely 30.0–40.0%, while the rate of recurrence is up to 50.0% in 2 years (Thomas and Zhu, 2005). Chemotherapy could not significantly suppress the progression of advanced HCC due to the low efficacy of chemotherapeutic drugs and severe side effects (Sun et al., 2019; Wang et al., 2019). Thus, molecular targeted therapy becomes the first-line and second-line treatment in clinical HCC therapy (Llovet et al., 2018), among which sorafenib (SOR), an oral multi-kinase inhibitor, was demonstrated to improve the overall survival as a kind of first-line treatment (Llovet et al., 2008). Lenvatinib (LEN), a new inhibitor of multiple receptor tyrosine kinases by oral administration, such as vascular endothelial growth factor (VEGF) receptors one to three, fibroblast growth factor (FGF) receptors one to four, platelet-derived growth factor (PDGF) receptor α, RET, and KIT, was approved as a preferred drug for the therapy of HCC with better antitumor efficacy than SOR (Kudo et al., 2018; Zhao et al., 2020). However, the side effects of these molecular targeted drugs hinder wide applications to some extent (Spallanzani et al., 2018; Zheng et al., 2021). To be specific, patients who apply LEN suffer side effects, including fatigue, anorexia, diarrhea, weight loss, even nausea and vomiting, or muscle soreness. In severe cases, headache, abdominal pain, and hand-foot syndrome will occur (Kaur et al., 2021; Otani et al., 2021; Platini et al., 2021). Moreover, due to its hydrophobicity, oral administration as the only option induces short half-life (Xu et al., 2021). Besides these side effects, the high cost and ease to generate resistance further limit the extensive use of molecular targeted drugs (Liu et al., 2021; Sun et al., 2021).

With the vigorous progression of emerging nanotechnology, nanoparticles with excellent biocompatibility and biodegradability, reduced side effects, prolonged retention time in the blood, and upregulated aggregation in the tumor tissue through the enhanced permeability and retention (EPR) effect displayed broad prospects (Liu et al., 2014; Bjornmalm et al., 2017; Liu et al., 2019; Wang et al., 2019). For HCC therapy, numerous preclinical studies have confirmed that the nanoplatforms exhibited lower side effects and improved efficacy than free drugs, indicating their great potentials for clinical applications in the future (Ayer and Klok, 2017; Shi et al., 2017; Jin et al., 2019; Wang et al., 2019). Furthermore, smart nanoparticles decorated with moderate radical groups exhibited tumor microenvironment-responsive characteristics, which could achieve precise delivery and release of antitumor agents (Huo et al., 2017; Chen et al., 2019; Zhang et al., 2020).

The microenvironment of HCC includes hypoxia (Ling et al., 2020), low pH (Zhang et al., 2016), and a high concentration of glutathione (GSH) (Tang et al., 2019). It should be noted that the GSH concentration is 50–1,000 times higher in the intracellular conditions compared with the level in the extracellular microenvironments (Huang et al., 2015; Feng et al., 2021b). In view of the background, this reduction-responsive nanoparticles were developed for targeted drug delivery (Feng et al., 2021a; Monteiro et al., 2021). As for HCC, the difference in GSH concentration is significant between the intracellular and extracellular microenvironments (Wang et al., 2019; Yang et al., 2021). The high GSH concentration in the tumor cells provides the possibility for the effectively disulfide bond (S−S)-contained nanogel for reduction-responsive delivery of therapeutic agents in HCC treatment.

In this work, our group developed methoxy poly(ethylene glycol)−poly(l-phenylalanine-*co*-l-cystine) (mPEG−P(LP-*co*-LC)) nanogel loading LEN in the hydrophobic core (NG/LEN) for HCC therapy. The nanogel could achieve concise delivery of LEN *via* the reduction-responsive characteristic of LC. NG/LEN enhanced the efficacy of tumor inhibition and down-regulated the systemic toxicity toward both subcutaneous and orthotopic HCC-allografted mice. The results confirmed that the reduction-responsive polypeptide nanogel was proven to be a good design to deliver molecular targeted drugs with enhanced effect for inhibition of tumor progression.

## Materials and Methods

### Cell and Animal Proposals

The rodent HCC cell line H22 cells were obtained from the National Collection of Authenticated Cell Culture (Shanghai, P. R. China) and incubated in the peritoneal cavity of BALB/c mice. The BALB/c mice aging 5 weeks and weighing 17.0 ± 1.5 g were purchased from the Charles River Laboratories (Beijing, P. R. China). All the mouse assessments were dealt with following the Guidelines for Animal Care and Use of Jilin University.

### *In vivo* Antitumor Efficacy and Safety Assays

The mouse H22 HCC-inoculated BALB/c mice were prepared by the injection of 100.0 μl of H22 cells including 1×10^6^ cells to the subcutaneous region in the armpit of the right anterior limb. As the increase of inoculated tumor volume to ∼120.0 mm^3^, the tumor-xenografted mice were stochastically separated into four groups (*n* = 6). Free NG, Free LEN, and NG/LEN were i.v. administrated to the HCC-bearing rodent animal model with an LEN dose of 10.0 mg per kg body weight (mg [kg BW]^−1^). The mouse in the control group was administrated with pure PBS. PBS or LEN formulations was administrated every 2 days, and six times was performed throughout the antitumor experiment *in vivo*. The tumor volume was detected every other day, and the body weight was monitored at the same frequency. Day 0 is the first day for antitumor therapy. On day 14 after the first injection, the mice were euthanized. After treatments, the tumor tissue and organs, i.e., the heart, liver, spleen, lung, and kidney, were isolated for safety assistance of treatment. The tumor size was assessed through Eq. 1.

$$V\left(mm^{3}\right)=\frac{L\times S^{2}}{2}$$ (1)

We constructed the orthotopic model of HCC in BALB/c mice according to the published protocol by the previous literature (Ma et al., 2014). Briefly, 5-week-old mice were shaved and anesthetized with 2% sodium pentobarbital (2.0 mg ml^−1^). The abdomen of the mouse was disinfected, and a 1 cm incision parallel to the right rib margin was performed to let the liver expose. And then, the 5 × 10^5^ collected H22 cells in 50.0 μl of PBS were inoculated into the left liver lobe, and 4−0 surgical sutures were used to suture the abdomen. Antibiotics were applied to prevent infection postoperatively. After 7 days of tumor injection, the rodent orthotopic HCC-allografted animals were stochastically separated into four groups (*n* = 6 for each group). These groups were PBS as a control, free NG, free LEN, and NG/LEN. The dose of LEN is 10.0 mg (kg BW)^−1^. Every 2 days, PBS of the formulations was administrated into the tail vein of tumor-bearing animals for treatment, and five times therapy was performed in the antitumor experiment *in vivo*.

### Histopathological Assays

After all the treatments, all the tumor-bearing animals were euthanized. The organs, including the heart, lung, liver, spleen, and kidney, were isolated and fixed in 4.0% (*W*/*V*) paraformaldehyde (PFA) for 1 day. Then, the samples were dehydrated, cleared, infiltrated, and embedded by wax, and cut into 5-μm thick slices. After that, the prepared slices were stained by hematoxylin and eosin (H&E) staining for histopathological assessments.

## Results and Discussion

### Synthesis of Methoxy Poly(ethylene glycol)−Polypeptide Nanogel and Its Characterizations

**FIGURE 1:**
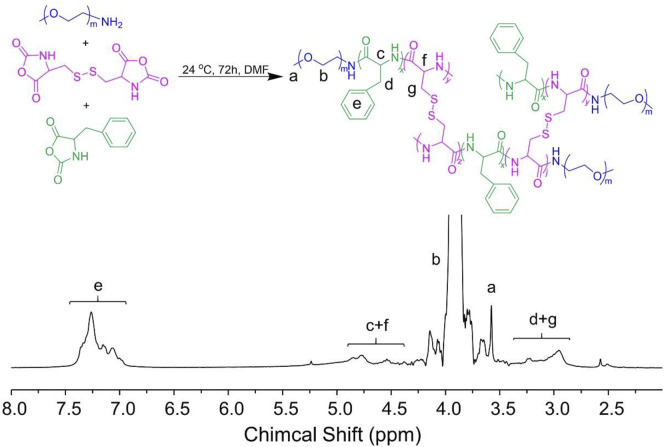
^1^H NMR result of mPEG−P(LP-*co*-LC).

**FIGURE 2:**
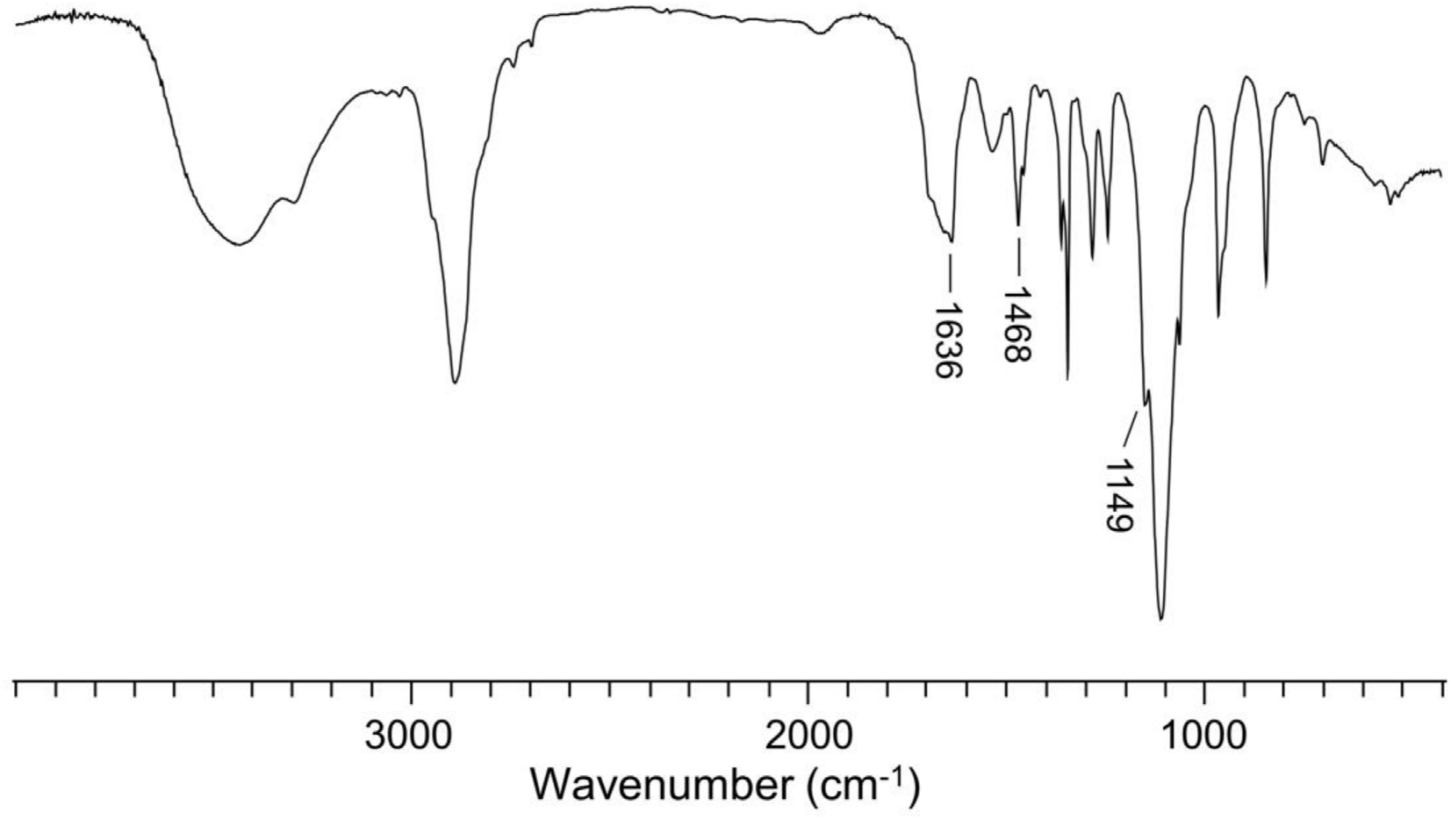
FT-IR result of mPEG−P(LP-*co*-LC).

The S−S-crosslinked mPEG−polypeptide nanogel was constructed by a hydrophobic reduction-responsive S−S-contained core of P(LP-*co*-LC) and a hydrophilic shell of mPEG. Briefly, mPEG−P(LP-*co*-LC) was prepared by the one-step ROP of LP NCA and LC NCA using mPEG-NH_2_ as an initiator. The molecular structure of mPEG−P(LP-*co*-LC) was first detected by proton nuclear magnetic resonance (^1^H NMR) and Fourier-transform infrared spectroscopy (FT-IR), as shown in Figures 1, 2, respectively. As depicted in Figure 1, the ^1^H NMR spectrum displayed the signal at 7.26 ppm (e), which should be assigned to the proton resonance in benzene ring of the LP unit (C_6_ *H*_*5*_−, 5H). The peaks at 4.85−4.31 ppm (c + f) should be attributed to the proton resonance of the polypeptide backbone (−C(O)C*H*(CH_2_C_6_H_5_)NH−, 1H and −C(O)C*H*(CH_2_S−)NH−, 1H). The signals at 3.90 (b) and 3.58 ppm (a) should be assigned to the resonance of the methylene and end methoxyl protons in mPEG (−C*H*_*2*_C*H*_*2*_−, 4H and C*H*_*3*_−, 3H), respectively. The methylene protons in the side groups of polypeptide provided the characteristic signals at 3.26−2.94 ppm (d + g) (C_6_H_5_C*H*_*2*_−, 2H and −SC*H*_*2*_−, 2H).

As shown in Figure 2, the FT-IR spectrum of mPEG−P(LP-*co*-LC) was further demonstrated the molecular structure of mPEG−polypeptide through the presence of typical absorptions at 1,636 (ʋ_C=O_) and 1,468 cm^−1^ (ʋ_C(O)–NH_), assigned to the amide bond in the backbone of polypeptide backbone, and 1,149 cm^−1^ (ʋ_C−O−C_) attributed to the ether bond in mPEG.

### Preparation and Characterizations of Lenvatinib-Loaded Nanogel

**FIGURE 3:**
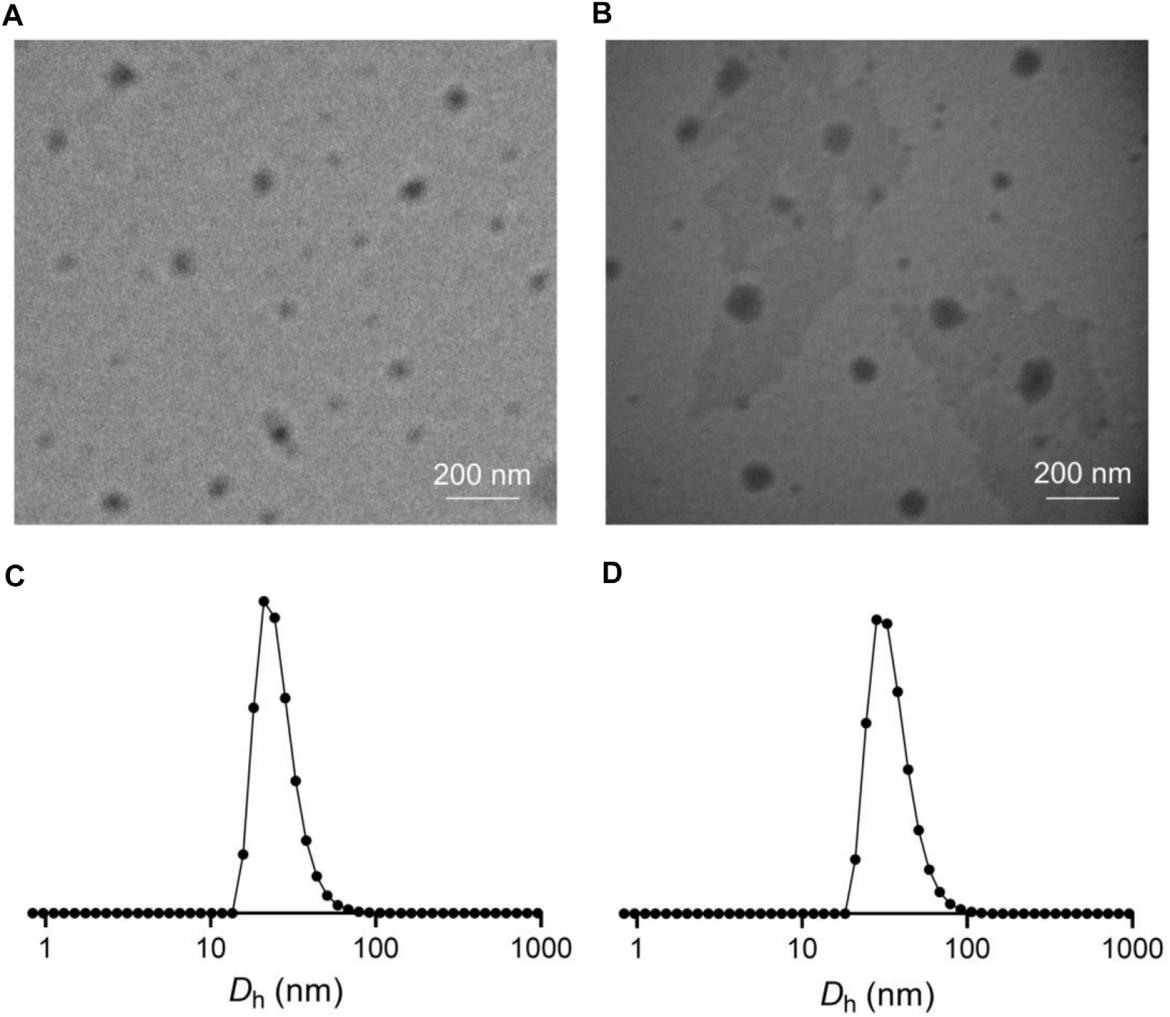
Typical TEM images and *D*_h_s of NG **(A,C)** and NG/LEN **(B,D)**. *D*_h_s detected by DLS were preceded by intensity mode.

**FIGURE 4:**
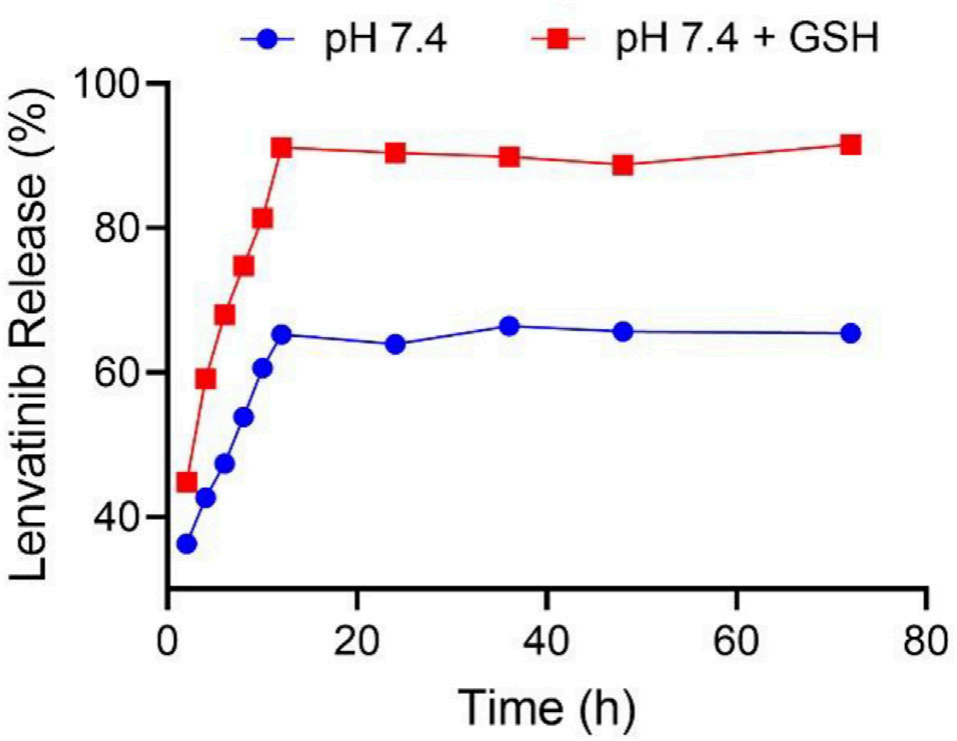
NG/LEN released LEN at the presence or absence of 10.0 mM GSH in pH 7.4 PBS. Each set of data is represented as mean ± SD (*n* = 3).

The LEN-loaded mPEG−polypeptide nanogel NG/LEN was obtained through a protocol of dispersion and dialysis (Yang et al., 2021). The drug loading content (DLC) and drug loading efficiency (DLE) of intracellular reduction-responsive NG/LEN were calculated to be 8.0 and 48.0%, respectively. The size and morphology of NG/LEN were detected by transmission electron microscopy (TEM) and dynamic laser scattering (DLS). The results of sizes and their distribution were displayed in Figure 3. As depicted in Figures 3A,B, both LG and NG/LEN showed as spheres with an apparent size of about 63 and 80 nm, respectively (Figure 4). The hydrodynamic diameters (*D*_h_) of NG and NG/LEN were detected to be 81 and 96 nm, respectively, as revealed in Figure 3B. The diameter distribution of NG/LEN was beneficial to passive tumor targeting through the EPR effect (Ding et al., 2019).

The drug loading properties are critical for nanoparticles as nanocarriers of drugs (Ferrari et al., 2018). The controlled drug release behaviors of nanomedicines are also crucial for targeted drug delivery during blood circulation and in the cancer cell microenvironments to improve the tumor inhibition efficacy and decrease the systemic toxicity (Wei et al., 2021). The intracellular level of GSH of the cancer cells is detected to be ten times higher than the extracellular concentration in the tumor microenvironments (Torchilin, 2014). As shown in Figure 4, in the medium of PBS without GSH, the accumulative release amount of LEN from NG/LEN was 65.4% in 12 h. The presence of PBS significantly accelerated the release of LEN from laden mPEG−polypeptide nanogel. At the same time point, the accumulative amount of LEN released from laden nanogel was increased to 91.6% in the mimic intracellular microenvironments, i.e., PBS with 10.0 nM GSH. The results demonstrated that NG/LEN could selectively deliver the molecular targeted drug LEN to the cells (Feng et al., 2021b).

### Antitumor and Safety Evaluation of Laden Nanogel Toward Subcutaneous Hepatocellular Carcinoma Model

The appropriate properties of NG/LEN, including morphology, size, and GSH-responsive drug release behavior, and so forth, laid a solid foundation for tumor inhibition *in vivo*. To evaluate the antitumor efficacy of NG/LEN, a subcutaneous HCC model was constructed by injection of 1 × 10^6^ H22 cells for each mouse in the subcutaneous region of armpit in right anterior limb. When the growth of tumor increased to about 120 mm^3^, the subcutaneous HCC-allografted model animals were stochastically separated into five groups and treated with PBS, free NG, LEN, or NG/LEN every 2 days for six times. The dose of LEN was set as 10.0 mg (kg BW)^−1^.

**FIGURE 5:**
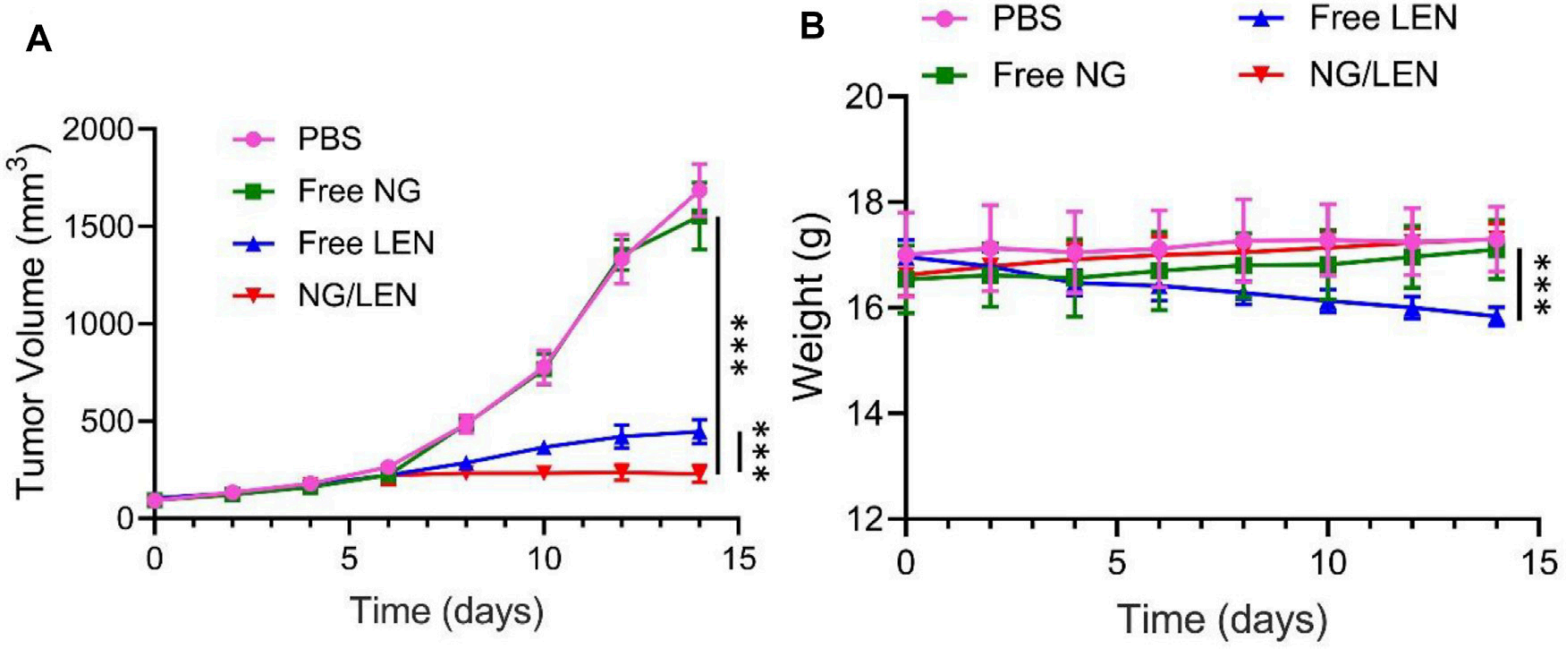
**(A)** *In vivo* tumor inhibition efficacies of PBS, free NG, free LEN, or NG/LEN on mouse model of subcutaneous HCC tumor model and **(B)** corresponding body weight change during treatment. Each set of data is represented as mean ± SD (*n* = 6; ***p < 0.001).

**FIGURE 6:**
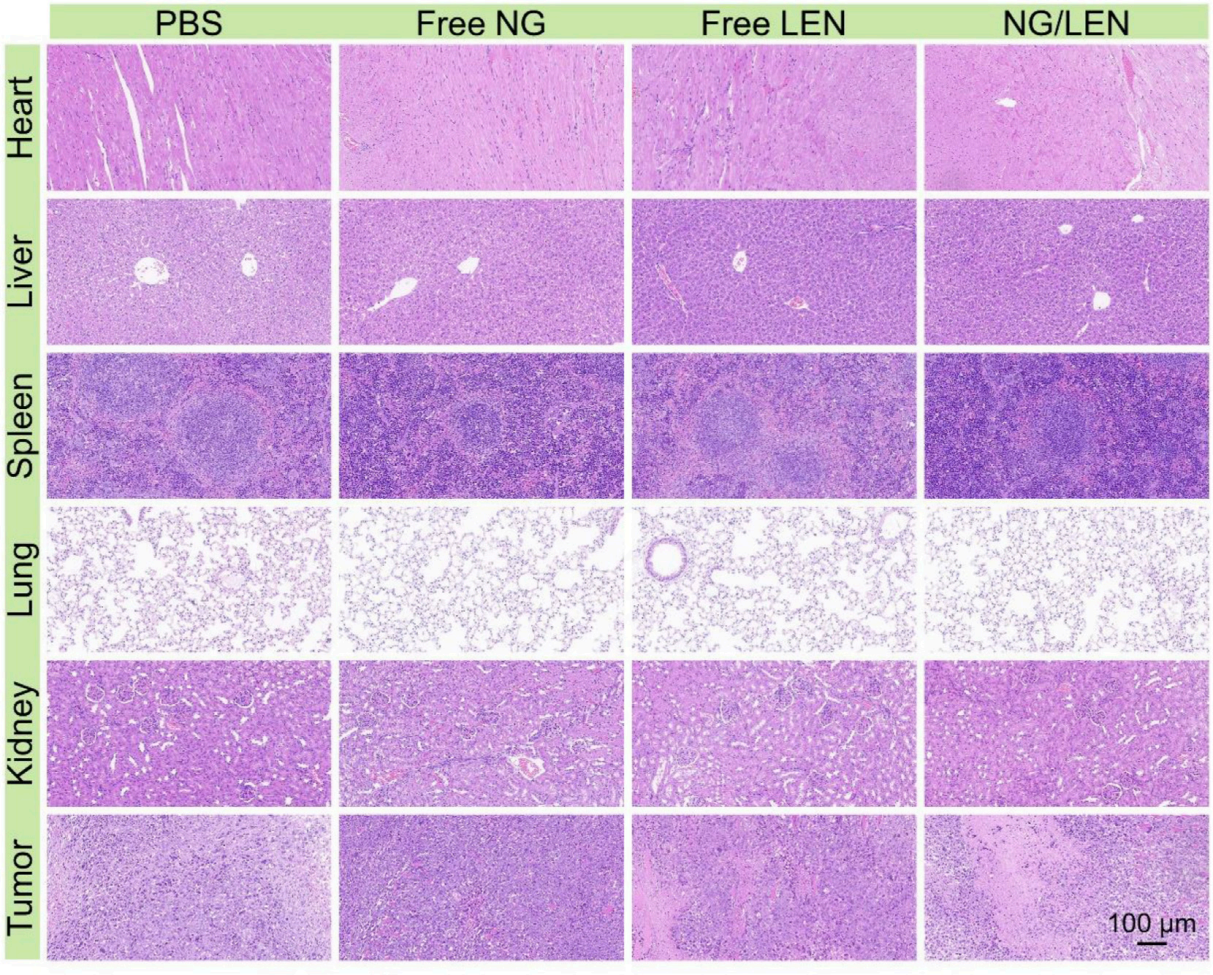
Histopathological analysis of organ sections, i.e., heart, liver, spleen, lung, kidney, and liver tumor after treatments of PBS, free NG, free LEN, or NG/LEN toward subcutaneous HCC tumor model.

As revealed by Figure 5A, NG/LEN showed the best antitumor efficacy, and only a slight change of tumor volume from ∼120 to 230 mm^3^ after the treatment with NG/LEN was observed in 14 days. Free LEN could also suppress the HCC growth, and the tumor grew to about 450 mm^3^ in the free LEN group during the experimental period. Free NG did not affect the HCC growth. After the treatment with free NG, the tumor volume was detected to be around 1,600 mm^3^, which was the same as that of PBS as a control. Furthermore, the best tumor inhibition efficiency of NG/LEN was demonstrated by the biggest necrotic area in the tumor section treated by NG/LEN (Figure 6). The results showed that LEN could effectively inhibit the HCC progression, and the encapsulation by mPEG−polypeptide nanogel could improve the efficacy of LEN further.

In addition to the excellent antitumor efficacy, the negligible side effects are another critical factor in the clinical application of nanomedicines (Zheng et al., 2021). To evaluate the safety of LEN formulations, the change of body weight was monitored during the treatments with LEN formulations. As depicted in Figure 5B, no significant difference was observed for the detected body weight among the groups of control treated with PBS, free NG, and NG/LEN, while the body weight decreased significantly in the free LEN group. The results indicated that the presence of mPEG−PEGsence of nanogel could effectively reduce the side effects of LEN.

The injury of organs always induced a decrease in body weight. At the end of the antitumor experiment *in vivo* on day 14, the organs, including the heart, liver, spleen, lung, and kidney, were collected after euthanasia of tumor-bearing mice after treatments. After being fixed in 4.0% (*W*/*V*) PFA in 1 day, the organs were dehydrated, cleared, embedded in wax, and cut into 5-μm thick slices for H&E staining. As revealed by Figure 6, the toxicity of LEN toward the heart and liver was observed from the H&E-stained sections. After treatment with LEN, the arrangement of cardiomyocytes was disordered, and some muscle fibers were broken. In addition, the structure of hepatic lobules was irregular, and some hepatocytes were stained shallowly and slightly vacuolated. At the same time, no histopathological morphologies were observed in the sections of the spleen, lung, and kidney. Therefore, the reduced body weight of tumor-bearing mice treated with LEN should be attributed to heart and liver injuries.

### Antitumor and Safety Evaluation of Laden Nanogel Toward Orthotopic Hepatocellular Carcinoma Model

The orthotopic HCC model is more meaningful to detected the tumor inhibition efficiency of different LEN medicines because the microenvironments of tumor for the orthotopic model is more close to the clinical one (Wang et al., 2019). In this study, the orthotopic HCC-allografted mice were constructed by the inoculation of H22 cells into the left liver lobe. On day 7 post-injection, the tumor-bearing animals were stochastically separated into four groups and treated with PBS, free NG, free LEN, or NG/LEN every 2 days for five times. The dose of LEN was also set as 10.0 mg (kg BW)^−1^.

**FIGURE 7:**
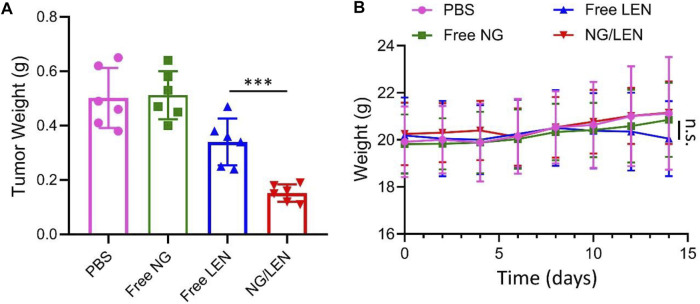
**(A)** *In vivo* tumor inhibition efficacies of PBS, free NG, free LEN, or NG/LEN on orthotopic model of HCC in BALB/c mice and **(B)** corresponding body weight change during treatment. Each set of data is represented as mean ± SD (*n* = 6; ***p < 0.001).

**FIGURE 8:**
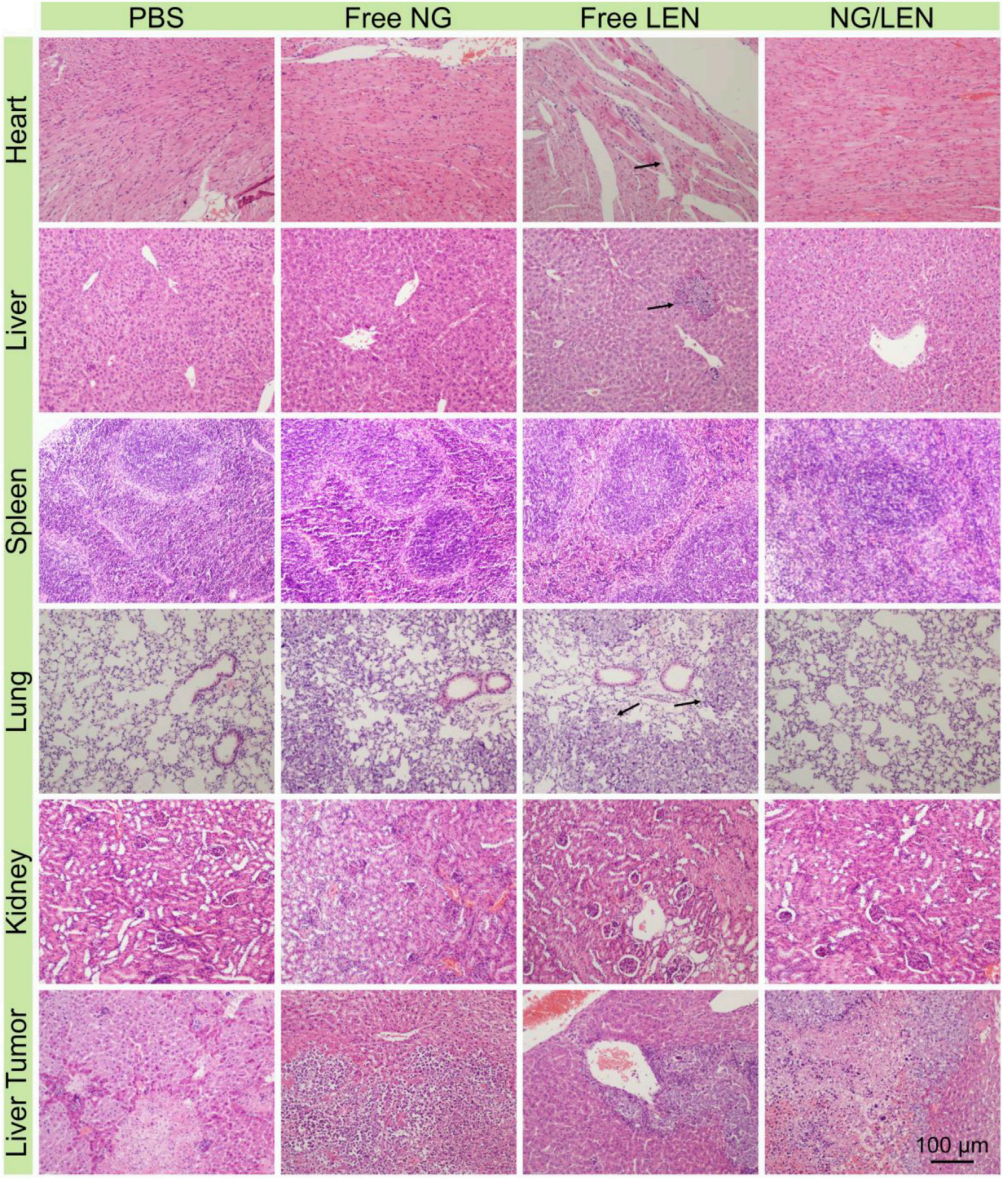
Histopathological analysis of organ sections, *i.e.*, heart, liver, spleen, lung, kidney, and liver tumor after treatment with PBS, free NG, free LEN, or NG/LEN toward orthotopic HCC model of BALB/c mice. Arrows indicate the injury parts.

Among all treatments, the NG/LEN group exhibited the smallest tumor weight of 0.15 g, while the groups of LEN, free NG, and PBS as a control showed 0.34, 0.51, and 0.5 g, respectively (Figure 7A). The results indicated that the nanosized LEN also exhibited the best antitumor efficacy toward the orthotopic HCC mouse model. The excellent antitumor capability of NG/LEN was confirmed by the most extensive region of tumor tissue necrosis detected from the H&E-stained slice of tumor tissue (Figure 8).

The safety of treatment was revealed by changing body weight and the H&E-stained tissue slices of main organs, i.e., the heart, liver, spleen, lung, and kidney. As demonstrated by the result of Figure 7B, no significant body weight change was monitored for the animals treated with all the LEN formulations, indicating the excellent safety of NG/LEN. However, the weight loss trend was observed in the LEN group from day 12, which revealed the toxicity of LEN to the tumor-bearing mice. In addition, the toxicity of LEN to the heart, liver, and lung was demonstrated by the H&E-stained tissue sections of organ tissues. As revealed by Figure 8, the mouse treated with LEN demonstrated the sign of liver injury. For the liver tissue section of the LEN group, the structure of hepatic lobules was irregular, the volume of hepatocytes increased, the cytoplasm was loose, the color became light, mild vacuole-like morphology emerged, and punctate necrosis was accompanied. In addition, the disordered arrangement of cardiomyocytes and broken muscle fibers were observed from the heart in the LEN group. There were many inflammatory cells and cellulose exudate in the lung of the LEN group. The results revealed that LEN exhibited significant toxicity toward the heart, liver, and lung of the tumor-bearing mice. At the same time, the encapsulation by mPEG−polypeptide nanogel could significantly decrease the toxicity of LEN along with the enhanced antitumor efficacy.

## Conclusion

Molecular targeted therapy is the first-line treatment modality for advanced HCC. In this study, a S−S-crosslinked mPEG−P(LP-*co*-LC) nanogel was prepared by the ROP of hydrophobic or reduction-responsive amino acid NCA monomers. The first-line molecular targeted drug LEN could be encapsulated into the mPEG−polypeptide nanogel through dispersion and dialysis. NG/LEN showed appropriate size (<100 nm) for HCC-targeted LEN delivery through the EPR effect. More importantly, NG/LEN demonstrated excellent antitumor efficacy toward both subcutaneous and orthotopic HCC models with reduced side effects. Therefore NG/LEN exhibited promising potential for HCC therapy in clinic.

## Data Availability

The raw data supporting the conclusion of this article will be made available by the authors, without undue reservation.
