# Supplementary material for: Intracellular Reduction-Responsive Molecular Targeted Nanomedicine for Hepatocellular Carcinoma Therapy
Source: Front Pharmacol. 2022 Jan 10;12:809125. doi: 10.3389/fphar.2021.809125 (PMC8784786; doi:10.3389/fphar.2021.809125)
Supplement: Supplementary file 1 [file DataSheet1.docx]

Supplementary Material

Intracellular Reduction-Responsive Molecular Targeted Nanomedicine for Hepatocellular Carcinoma Therapy

Lei Ding^1^, Ping Zhang^1^*, Xu Huang^1^, Kunmeng Yang^1^, Xingkai Liu^1^ and Zhenxiang Yu^2^*

^1^Department of Hepatobiliary and Pancreatic Surgery, The First Hospital of Jilin University, 1 Xinmin Street, Changchun 130021, P. R. China

^2^Department of Respiration, The First Hospital of Jilin University, 1 Xinmin Street, Changchun 130021, P. R. China

***Correspondence:**

Ping Zhang

z_ping@jlu.edu.cn

Zhenxiang Yu

yuzx@jlu.edu.cn

# Materials and methods

## Materials

LEN mesylate was purchased from Beijing Huafeng United Technology Co., Ltd. (Beijing, P. R. China). L-phenylalanine and L-cystine were purchased from D & B Technology Co., Ltd. (Shanghai, P. R. China). mPEG (*M*_n_ = 5000 g mol^−1^) was purchased from Sigma-Aldrich (Shanghai, P. R. China). Clear 6-well and 96-well tissue culture polystyrene plates were purchased from Corning Costar Co. (Cambridge, MA, USA). The deionized water was prepared through Milli-Q water purification equipment (Millipore Co., Billerica, MA, USA).

## Synthesis of mPEG−polypeptide nanogel

According to the previously reported protocol, the amino-terminated mPEG (mPEG-NH_2_) was synthesized (Thompson et al., 2008). mPEG5k−P(LP_10_-*co*-LC_5_) nanogel was synthesized through one-step ring-opening polymerization (ROP) of L-phenylalanine *N*-carboxyanhydride (LP NCA) and L-cystine *N*-carboxyanhydride (LC NCA) following the previously reported proposal (Ding et al., 2011;Huang et al., 2015). Briefly, L-Phe NCA (0.85 g), L-Cys NCA (0.65 g), and mPEG-NH_2_ (2.22 g) were dissolved in 30.0 mL of dry *N*,*N*-dimethylformamide (DMF) in a flame-dry ampoule for three days at room temperature. The solution was precipitated by diethyl ether. The precipitation was washed by a small amount of diethyl ether thrice and dried under vacuum at room temperature.

## Characterizations

The contents of carbon, hydrogen, nitrogen, and sulfur elements in mPEG−P(LP-*co*-LC) were measured by elemental analysis (Vario EL III, Elementar, Germany). Proton nuclear magnetic resonance (^1^H NMR) spectra were recorded in trifluoroacetic acid-*d* (TFA-*d*) on Bruker AV 300 NMR spectrometer (Bruker Biospin GmbH, Rheinstetten, Germany). Fourier-transform infrared spectroscopy (FT-IR) spectra were conducted on a Bio-Rad Win-IR instrument (Bio-Rad, Digitlab Division, Cambridge, MA, USA) using the potassium bromide (KBr) method. Transmission electron microscopy (TEM) measurements were performed on a JEOL JEM-1011 transmission electron microscope (JEOL, Tokyo, Japan) with an accelerating voltage of 100 kV to detect the morphology and hydration size. Dynamic laser scattering (DLS) and zeta potential measurements were performed on a Malvern Zetasizer Nano ZS 90 (Malvern Instruments Ltd., Worcestershire, UK).

## *In vitro* drug loading and release

mPEG−P(LP-*co*-LC) nanogel (10.0 mg) and LEN mesylate (2.0 mg) were mixed in 1.0 mL of DMF for 2 h. Then, the mixture was added dropwise into 3.0 mL of deionized water under stirring. After that, the mixture was stirred at room temperature for 12 h, and the organic solvent was removed through dialysis for 12 h to get NG/LEN. The drug loading content (DLC%) and the drug loading efficiency (DLE%) of LEN-loaded nanogel were calculated through the following Equations (1) and (2):

$$DLC\%=\frac{Amount of loaded LEN}{Amount of LEN-loaded nanogel} (1)$$

$$DLE\%=\frac{Amount of loaded LEN}{The total amount of dissolved LEN} (2)$$

The *in vitro* LEN release behavior from NG/LEN was investigated in phosphate-buffered saline (PBS). The freeze-dried NG/LEN (1.0 mg) was suspended in 100.0 mL of PBS at pH 7.4 with or without 10.0 mM GSH and transferred into a dialysis bag (molecular weight cut-off (MWCO) = 3.5 kDa). The filled dialysis bag was placed into a beaker with 100.0 mL of PBS. Then, the beaker was put into an oscillation box at 37 °C with continuous vibration at 70 revolutions per minute (rpm). 2.0 mL of the external release medium was taken out at predetermined points, and the same volume of fresh PBS was replenished. The amount of loaded and released LEN was measured by high-performance liquid chromatography (HPLC; Waters Corp., Milford, MA, USA). The mobile phases are acetonitrile and distilled water with 0.1% methane acid. The spectrophotometer is at 252 nm for detection.

## Statistical analysis

The data were displayed as mean ± standard deviation (SD). Statistical analysis was performed by Graphpad Prism 7 for Windows. **P* < 0.05, ***P* < 0.01, and ****P* < 0.001 were considered statistically significant.

# Reference

Ding, J., Shi, F., Xiao, C., Lin, L., Chen, L., He, C., Zhuang, X., and Chen, X. (2011). One-step preparation of reduction-responsive poly(ethylene glycol)-poly(amino acid)s nanogels as efficient intracellular drug delivery platforms. *Polymer Chemistry* 2**,** 2857-2864.

Huang, K., Shi, B., Xu, W., Ding, J., Yang, Y., Liu, H., Zhuang, X., and Chen, X. (2015). Reduction-responsive polypeptide nanogel delivers antitumor drug for improved efficacy and safety. *Acta Biomater* 27**,** 179-193.

Thompson, M.S., Vadala, T.P., Vadala, M.L., Lin, Y., and Riffle, J.S. (2008). Synthesis and applications of heterobifunctional poly(ethylene oxide) oligomers. *Polymer* 49**,** 345-373.
